# Phenotypic and genotypic characterization of near-isogenic lines targeting a major 4BL QTL responsible for pre-harvest sprouting in wheat

**DOI:** 10.1186/s12870-019-1961-1

**Published:** 2019-08-09

**Authors:** Xingyi Wang, Hui Liu, Guannan Liu, Md Sultan Mia, Kadambot H. M. Siddique, Guijun Yan

**Affiliations:** 1 UWA School of Agriculture and Environment and The UWA Institute of Agriculture, The University of Western Australia, Perth, WA 6009 Australia; 2 Plant Breeding Division, Bangladesh Agricultural Research Institute, Joydebpur, Gazipur, 1701 Bangladesh

**Keywords:** Pre-harvest sprouting, Near-isogenic lines, SNP assay, Plant height, Grain number per spike, Wheat

## Abstract

**Background:**

Resistance to pre-harvest sprouting (PHS) is one of the major objectives in wheat breeding programs. However, the complex quantitative nature of this trait presents challenges when breeding for PHS resistance. Characterization of PHS using near-isogenic lines (NILs) targeting major quantitative trait locus/loci (QTL/QTLs) can be an effective strategy for the identification of responsible genes and underlying mechanisms.

**Results:**

In this study, multiple pairs of NILs were developed and confirmed for a major QTL located on the 4BL chromosome arm that contributes to PHS resistance in wheat, using a combined heterogeneous inbred family method and a fast generation cycling system. Phenotypic characterization of these confirmed NILs revealed significant differences in PHS resistance between the isolines, where the presence of the resistant allele increased the resistance to sprouting on spikes by 54.0–81.9% (average 70.8%) and reduced seed germination by 59.4–70.5% (average 66.2%). The 90 K SNP genotyping assay on the confirmed NIL pairs identified eight SNPs on 4BL, associated with five candidate genes; two of the candidate genes were related to seed dormancy. Agronomic traits in the NIL pairs were investigated; both plant height and grain number per spike were positively correlated with PHS susceptibility.

**Conclusions:**

This study confirmed multiple pairs of NILs and identified SNPs between PHS isolines, which are valuable resources for further fine-mapping of this locus to clone the major genes for PHS resistance and investigate the possible functional regulation of these genes on important agronomic traits, such as plant height and grain number per spike.

## Background

Pre-harvest sprouting (PHS) is a phenomenon in which seeds germinate on the intact spike or on the mother plant before harvest. PHS is triggered when matured grains encounter rain or air humidity close to saturation. In wheat, PHS results in degradation of starches in the grain rendering the grain unsuitable for milling into flour [1]. PHS occurs in many countries including China, Australia, Canada, USA, Europe and Japan [2]. Worldwide losses to PHS have been estimated at $1.2 billion a year [3]. Therefore, resistance to PHS is an economically important trait for wheat breeding programs [4]. Documented methods to alleviate losses associated with PHS in wheat include the application of clove bud oil to seeds to inhibit seed germination [5], but breeding resistant varieties remains the most economically efficient method.

Resistance to PHS in crops is a complex trait, which is conditioned by both genetic and environmental factors [6–8]. PHS is due to insufficient grain dormancy during seed development and maturation [9] and its resistance can be affected by factors including substances in the glume that inhibit germination [10], physical barriers to water penetration in the spike [11], and spike morphology [12]. Therefore, germination testing directly on a spike (spike sprouting test) reflects PHS performance better than a standard seed germination test [13].

PHS resistance is a quantitative trait that is regulated by several genes or quantitative trait loci (QTLs) [14]. QTLs for PHS resistance have been reported on almost all chromosomes in bread wheat [14–16]; several major QTLs have been located on group 4 chromosomes with repeated detections in different studies [17–27]. The genes underlying the major PHS resistance QTL on 4AL have been recently identified [8]. Despite the considerable attention paid to the 4AL QTL, little research has been conducted to characterize other major QTLs on group 4 chromosomes. A QTL within the centromeric region of the long arm of chromosome 4B, *QPhs.ocs-4B.1*, located between markers *Xgwm495* and *Xgwm375*, was identified as a major QTL for grain dormancy in the medium-dormant wheat Chinese Spring, explaining about 20% of the phenotypic variation [19]. Kato et al. [17] identified three QTLs on group 4 chromosomes in doubled-haploid lines, of which *Qphs.ocs-4B.2*, located within the telomere region of 4BL, had consistent effects on seed dormancy in seeds produced in both glasshouse and growth chamber trials, and was homoeologous with a PHS QTL on 4D. Kumar et al. [25] located a major QTL, *QPhs.spa-4B*, explaining 35–60% of the phenotypic variation in field environments. Furthermore, using composite interval mapping, Lin et al. [26] detected a minor QTL on 4B, *Qphs.pseru-4B.1*, between markers *Xbarc20* and *Xwmc238*, for both PHS resistance and seed dormancy, which explained 6.3–8.7% of the phenotypic variance.

In the past two decades, QTLs on chromosome 4B have gained attention, as numerous studies have identified a ‘QTL-hotspot’ region on the chromosome responsible for many agronomic traits, especially plant height and yield-related traits [28–34]. For plant height, the *Rht-B1* gene on chromosome 4B has a profound impact on stem elongation and grain number per spike [29]. Marza et al. [31] consistently detected QTLs that influence plant height, spike length and grain number per spike on chromosome 4B in their nine experimental environments. Wang et al. [35] identified a major QTL for grain number per spike on chromosome 4B, and its contribution explained 18.2% of the phenotypic variation. Recently, Guan et al. [32] identified a novel QTL for the heat susceptibility index of thousand-grain weight on chromosome 4BL that explained ~ 10% of the phenotypic variation.

Development of near-isogenic lines (NILs) for major QTLs will facilitate detailed characterization of the QTLs, gene identification and functional analysis, and marker-assisted breeding of PHS resistance [36]. NILs are a pair of plants with a nearly identical genetic background except for a single section/locus on one chromosome. This means that any differences between the pairs will be due to differences in genes at the target locus, turning a complex quantitative trait into a simple Mendelian factor with predictable patterns of segregation and inheritance. Generating more than one NIL pair from different initial crosses ensures that the target gene/locus and its function are validated under different genetic backgrounds. Here, we report the development, confirmation, and genotypic and phenotypic characterization of five pairs of NILs targeting the major 4BL QTL, *QPhs.ocs-4B.1*, responsible for PHS resistance in wheat. Correlations between PHS and plant height, and grain number per spike were also investigated.

## Results

### Development of NILs for PHS resistance

**Fig. 1:**
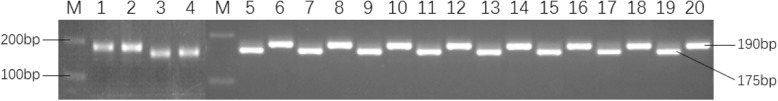
Agarose gel image of PCR products amplified from the parents and the confirmed isolines using maker *Xgwm495*. The 175 bp-fragments represent the resistant allele, while the 190 bp fragments represent the susceptible allele. M: 100 bp DNA ladder; numbers 1 to 20 refer to the four parents (Chara, Westonia, DM5637B*8 and SUN326AE) and the eight confirmed isoline pairs as indicated in Table 1

Molecular marker screening of the two cross-population lines with *Xgwm495* grouped the progeny into three distinct classes: homozygous with PHS resistant allele of 175 bp, homozygous with susceptible allele of 190 bp, and heterozygous with both 175 bp and 190 bp alleles. Heterozygous lines were selected from each generation for further advancement and marker screening until F_7_. The marker-assisted heterogeneous inbred family (HIF) method in the two populations generated 19 F_7_ heterozygous lines—seven from the SUN326AE × Westonia population and 12 from the Chara × DM5637B*8 population. From each heterozygous F_7_ line, two homozygous F_8_ isolines with contrasting alleles at marker *Xgwm495* were selected as a NIL pair. The 19 pairs of NILs were ranked for their PHS resistance following the sprouting test assay. Selected NIL pairs and their allele categories are shown in Fig. 1.

### Evaluation of NILs using sprouting test

**Fig. 2:**
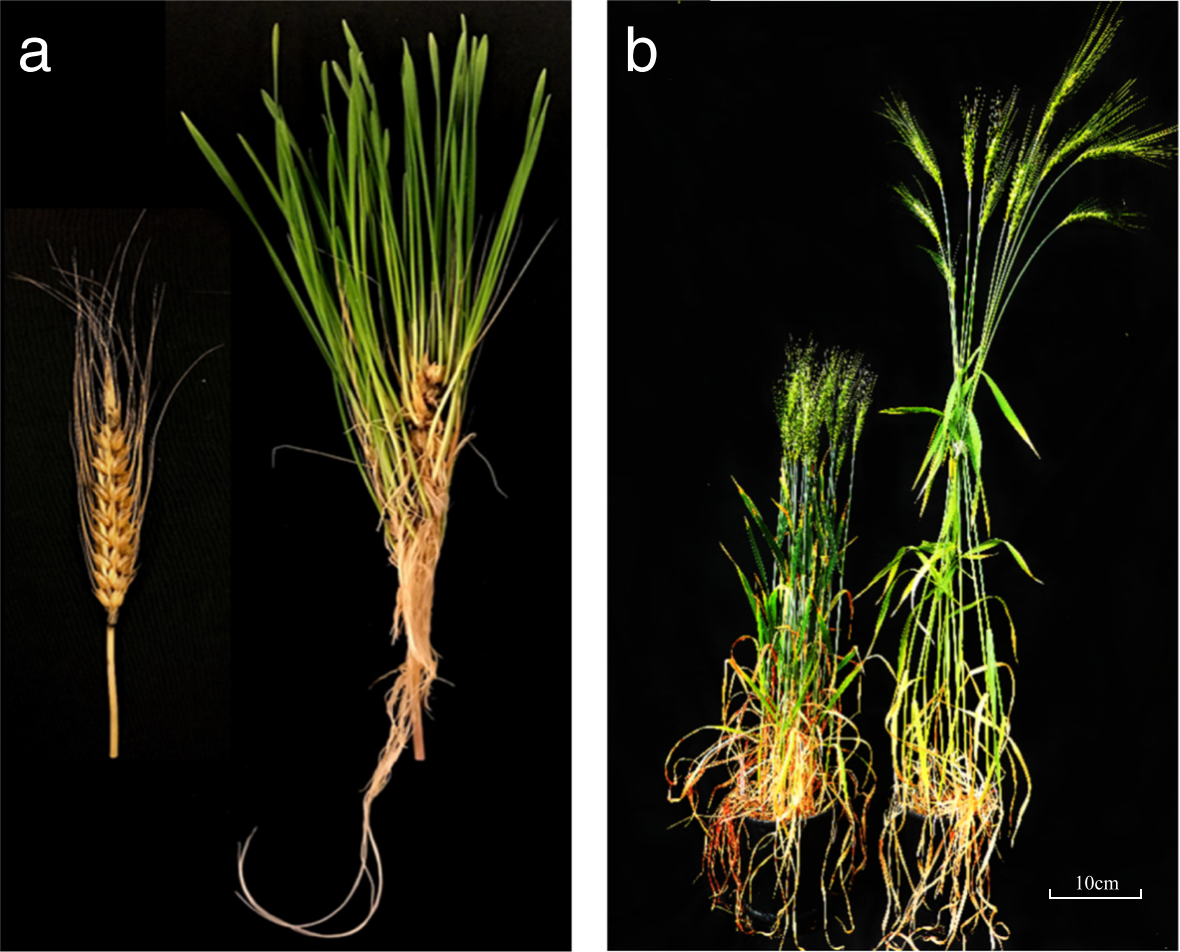
Spikes and plants of a pair of near-isogenic lines (NIL_PHSR4BL_5). The significant differences between the resistant (on the left of each image) and the susceptible (on the right of each image) isoline was observed in **a** spike sprouting on day 7, and **b** plant height

The sprouting test, using whole spikes, was conducted to analyze PHS resistance in the 19 putative NIL pairs. Differences in sprouting were observed between the resistant and susceptible isolines from day 3 of the test. For the susceptible spikes, young leaves and roots started to grow out of the wrapped filter paper. After removing the wrapped filter paper on day 7, numerous seedlings were observed on each spike of the susceptible isolines, but none or few were observed on each spike of the resistant isolines (Fig. 2). However, drying and threshing of the resistant isoline spikes detected some additional germinated seeds, as their seed coats were broken, and embryos started to grow. Statistical analysis showed significant differences in sprouting in eight of the 19 putative NIL isoline pairs (Table 1).

**Table 1:** Sprouting and germination tests of four parents and eight confirmed pairs of NILs for *QPhs.ocs-4B.1* conferring PHS resistance in wheat

Number	Parents/NILs	Genetic background	SP (%)	Difference in SP (%)	GI	Difference in GI (%)
1	Chara (Non-dormant, PHS susceptible parent)		68.26		0.63	
2	Westonia (Non-dormant, PHS susceptible parent)		83.40		0.74	
3	DM5637B*8 (Dormant, PHS resistant parent)		14.85		0.18	
4	SUN326AE (Dormant, PHS resistant parent)		19.31		0.22	
5	NIL_PHSR4BL_1R	SUN326AE × Westonia F8	21.44	70.86**	0.33	59.41**
6	NIL_PHSR4BL_1S		73.56		0.81	
7	NIL_PHSR4BL_2R	SUN326AE × Westonia F8	33.60	54.01**	0.29	61.56*
8	NIL_PHSR4BL_2S		73.05		0.76	
9	NIL_PHSR4BL_3R	Chara × DM5637B*8 F8	13.69	76.75**	0.69	10.84
10	NIL_PHSR4BL_3S		58.88		0.77	
11	NIL_PHSR4BL_4R	Chara × DM5637B*8 F8	16.19	69.61**	0.19	70.52*
12	NIL_PHSR4BL_4S		53.28		0.64	
13	NIL_PHSR4BL_5R	Chara × DM5637B*8 F8	14.71	77.60*	0.22	69.54**
14	NIL_PHSR4BL_5S		65.65		0.72	
15	NIL_PHSR4BL_6R	Chara × DM5637B*8 F8	21.18	58.90*	0.67	8.79
16	NIL_PHSR4BL_6S		51.53		0.73	
17	NIL_PHSR4BL_7R	Chara × DM5637B*8 F8	7.11	81.88**	0.19	70.11*
18	NIL_PHSR4BL_7S		39.22		0.65	
19	NIL_PHSR4BL_8R	Chara × DM5637B*8 F8	20.14	64.44*	0.67	6.31
20	NIL_PHSR4BL_8S		56.63		0.72	

‘R’ lines are those with an allele from the resistant parent (SUN326AE or DM5637B*8), and ‘S’ lines are those with an allele from the susceptible parent (Westonia or Chara); * and ** represent significant differences at *p* < 0.05 and *p* < 0.01 levels, respectively; ‘SP’ is sprouting percentage; ‘GI’ is germination index

Two of the eight confirmed NIL pairs—NIL_PHSR4BL_1 and NIL_PHSR4BL_2—were derived from the SUN326AE × Westonia population, and the remaining six—NIL_PHSR4BL_3, NIL_PHSR4BL_4, NIL_PHSR4BL_5, NIL_PHSR4BL_6, NIL_PHSR4BL_7, and NIL_PHSR4BL_8 were derived from the Chara × DM5637B*8 population.

In general, isolines possessing the allele from the resistant parents had lower sprouting percentages than their respective counterparts for all eight NIL pairs. Sprouting percentages of the NILs from SUN326AE × Westonia population ranged from 20 to 73%. Isolines with the resistant allele (NIL_PHSR4BL_1R and NIL_PHSR4BL_2R) had an average sprouting percentage of 27.5%, which was significantly lower than the isoline with the susceptible allele (73.3%). Similarly, the six NIL pairs derived from the Chara × DM5637B*8 population varied considerably in terms of sprouting percentage. Isolines with the susceptible allele had sprouting percentages ranging from 39.2–58.9% (average 54.2%), more than threefold higher than isolines with the resistant allele, (7.1–21.2%). Of the confirmed NIL pairs, NIL_PHSR4BL_2 had the least significant difference between isolines for sprouting percentage (54.0%), while NIL_PHSR4BL_7 had the greatest difference (81.9%).

### Evaluation of NILs using GI assay

Germination index (GI) assay was conducted to analyze seed dormancy in the eight putative NIL pairs that had significant differences between the resistant and susceptible isolines in the sprouting test. Assay observation showed that seeds from all the isolines started germination on the second day of the test. For the isolines with susceptible alleles, more than half of the seeds germinated within 2 days, and all seeds had germinated before day 5. In contrast, for the resistant alleles, most of the seed germination occurred evenly from day 2 to day 4; some of the seeds even germinated on the day 6 or 7. GI analysis indicated that five of the eight pairs showed significant differences in GI between the isolines (Table 1). Two (NIL_PHSR4BL_1 and NIL_PHSR4BL_2) of these five NIL pairs were derived from the ‘SUN326AE × Westonia’ population, and the remaining three pairs (NIL_PHSR4BL_4, NIL_PHSR4BL_5, and NIL_PHSR4BL_7) were from ‘Chara × DM5637B*8’ population. Without exception, isolines with the allele from the resistant parent, ‘SUN326AE’ or ‘DM5637B*8’, always had a significantly lower GI than their counterparts for all of the five pairs of NILs. The increase of seed dormancy received from the resistant allele from ‘SUN326AE’ ranged from 59.4–61.6% (average 60.5%) and from ‘DM5637B*8’ ranged from 69.5–70.5% (average 70.1%).

Of the eight NIL pairs with significant differences between resistant and susceptible isolines in the sprouting test, three pairs (NIL_PHSR4BL_3, NIL_PHSR4BL_6 and NIL_PHSR4BL_8) had similarly high GIs between the isolines (0.67–0.77, average 0.71).

### Correlations of PHS with other agronomic traits

**Fig. 3:**
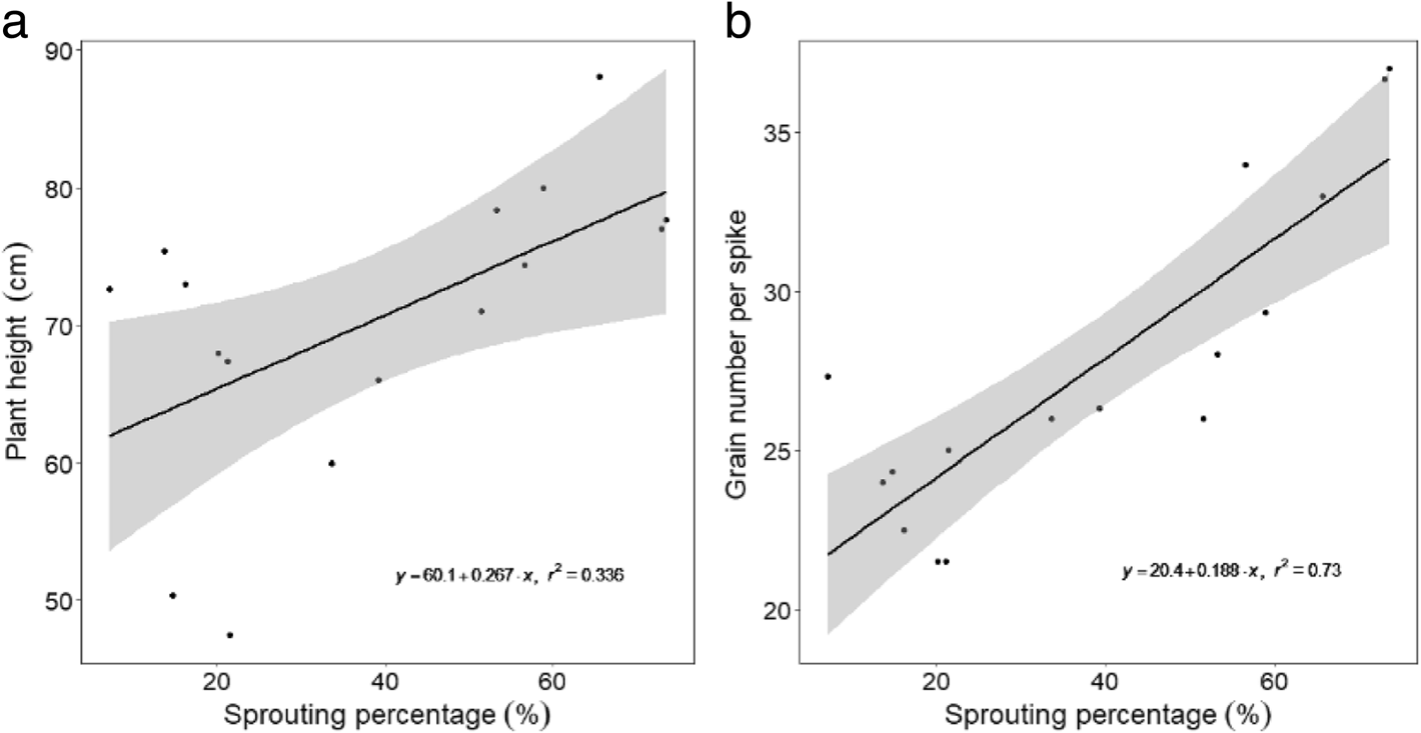
Correlation between sprouting percentage and **a** plant height, and **b** grain number per spike. All values are means of three replicates from the eight pairs of near-isogenic lines for PHS resistance

Of the 11 agronomic traits investigated, only two were significantly correlated with PHS. Figure 3 shows positive correlation in the eight confirmed NILs between sprouting percentage and plant height (*r*^*2*^ = 0.34, *p* < 0.05), and grain number per spike (*r*^*2*^ = 0.73, *p* < 0.001), but no significant correlations between sprouting percentage and the other nine traits.

**Fig. 4:**
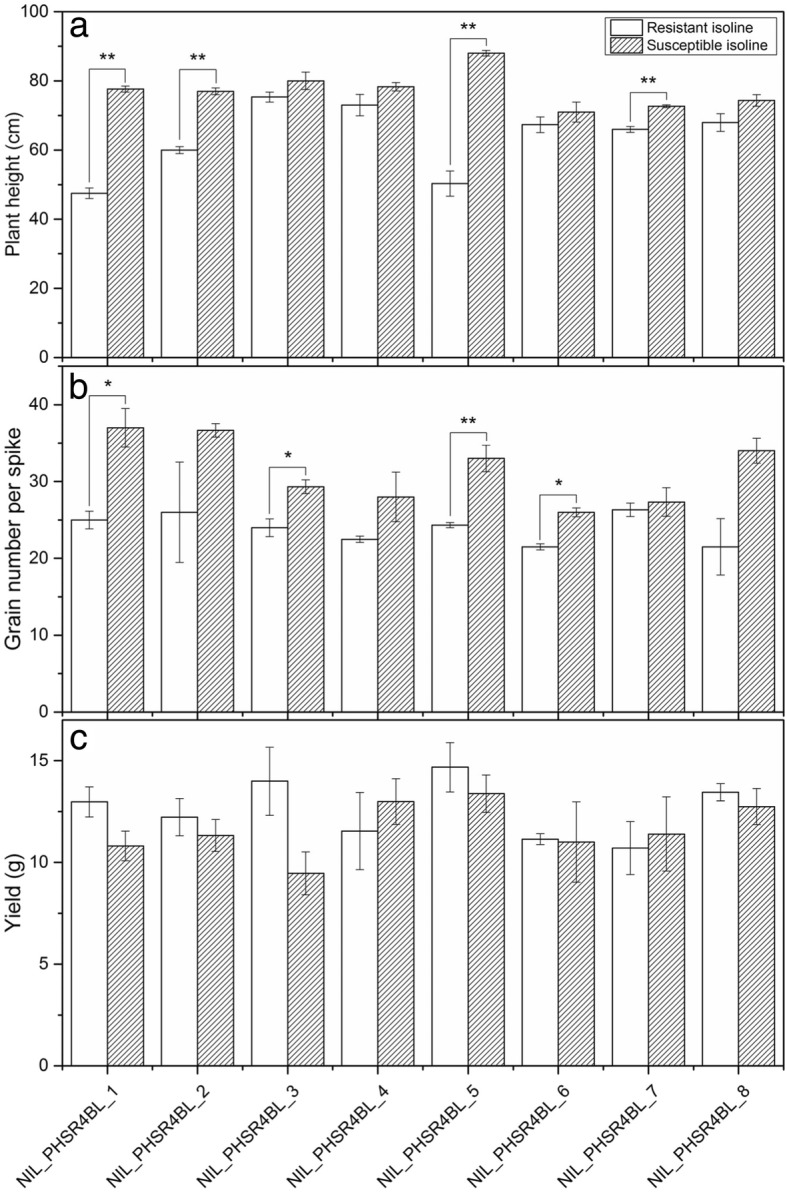
Differences in agronomic traits in the confirmed isolines with resistant and susceptible alleles. **a**: plant height, **b**: grain number per spike and **c**: yield per plant; ‘*’ and ‘**’ indicate significant differences between the isolines at *p* < 0.05 and *p* < 0.01, respectively

Further investigation showed significant differences between some of the NIL pairs for plant height and grain number per spike (Fig. 4). The resistant and susceptible isolines of four pairs of NILs, namely, NIL_PHSR4BL_1, NIL_PHSR4BL_2, NIL_PHSR4BL_5 and NIL_PHSR4BL_7, differed significantly (*p* < 0.01) for plant height (Fig. 2): the isolines with resistant allele were shorter than those with susceptible alleles. The NILs with resistant alleles, namely, NIL_PHSR4BL_1, NIL_PHSR4BL_3, NIL_PHSR4BL_5, and NIL_PHSR4BL_6, had significantly fewer grains per spike than those with susceptible alleles. Although the remaining NILs showed no significant differences between the resistant and susceptible isolines for plant height or grain number per spike, isolines with the susceptible allele were generally taller or produced more grains per spike than those with the resistant allele.

### SNP genotyping analysis

Five confirmed NIL pairs, with significant differences between resistant and susceptible isolines in both the sprouting test and GI assay, were subjected to 90 K iSelect genotyping assay. The 90 K iSelect genotyping assay was developed based on hexaploid wheat and *Aegilops tauschii* sequences [37]. Of the 81,587 SNPs on the array, 31,807 were analyzed across the 21 chromosomes after removing those that did not meet selection criteria and those without polymorphism. A total of 989 SNPs were assigned on the long arm of chromosome 4B according to the assembly results from Wang et al. [37]. Analyses of the 989 SNPs among the five confirmed pairs of isolines found that only eight SNPs, which were all located within the 4BL QTL region, showed consistent contrasting genotypes (AA or BB) between the resistant or susceptible isolines. Of the eight SNPs, BS00068851_51 was located between two genes TraesCS4B01G434700LC and TraesCS4B01G434800LC at their untranslated regions (UTRs); BS00096604_51 and IAAV5117, Ra_c27465_569 and Ra_c27465_564 were blasted on the same candidate genes TraesCS4B01G239700 and TraesCS4B02G253300, respectively. Therefore five candidate genes were positioned within the region of *QPhs.ocs-4B.1* by blasting the reference Chinese Spring genome (https://triticeaetoolbox.org/wheat//) (Table 2). Functional annotation of these genes indicated that *TraesCS4B01G239700* was a heat shock transcription factor and *TraesCS4B02G247900* was a DEAD-box ATP-dependent RNA helicase gene, which have been reported to be related to PHS resistance [38–42]. The remaining three genes *TraesCS4B02G253300*, *TraesCS4B02G217800* and *TraesCS4B02G229600* were involved in other metabolic processes.

**Table 2:** Annotation of candidate genes

Candidate genes	SNPs	Functions
TraesCS4B01G239700	BS00096604_51 / IAAV5117	Heat shock transcription factor
TraesCS4B02G253300	Ra_c27465_569 / Ra_c27465_564	Alpha-L-fucosidase 2
TraesCS4B02G217800	wsnp_Ra_c1992_3876325	myosin-binding protein (protein of unknown function, DUF593)
TraesCS4B02G247900	Ra_c3117_2098	DEAD-box ATP-dependent RNA helicase
TraesCS4B02G229600	Tdurum_contig69405_332	Oxidoreductase, 2OG-Fe(II) oxygenase family protein

## Discussion

In this study, we successfully produced and confirmed five pairs of NILs targeting *QPhs.ocs-4B.1,* a major QTL on chromosome 4BL conferring both PHS resistance and seed dormancy in wheat. This outcome was achieved through molecular screening and generation advancement of 240 genotypes from two different populations. We also identified two putative candidate genes of this locus that are related to seed dormancy. Characterization of these NIL pairs revealed that the presence of the allele from the resistant source, either SUN326AE or DM5637B*8, significantly increased PHS resistance. The large differences in PHS resistance and seed dormancy between the isolines of the five NIL pairs developed from two different genetic backgrounds further confirmed the significance of this major QTL in PHS resistance. These NIL pairs with confirmed marker-gene linkage are valuable materials for future cloning and functional studies of the gene(s) underlying the 4BL QTL for PHS resistance, development of functional markers that can be reliably used for marker-assisted selection, and investigation of the mechanisms for the correlations between PHS with plant height and grain number per spike.

In this study, two different methods were used to phenotype the putative NIL pairs—the sprouting test using intact spikes for direct PHS resistance and the GI for seed dormancy. The sprouting test confirmed eight pairs of NILs with significant differences between the isolines; five were further confirmed by GI for seed dormancy, while remaining three pairs had a similar high GI rate among the isolines. These results agree with previous reports that PHS resistance is a complex trait [6–8, 14]; while seed dormancy is a major genetic factor of PHS resistance, other factors could be involved in the overall PHS resistance such as glume substances that inhibit germination [10], physical barriers to water penetration in the spike [11] and spike morphology [12]. The three NIL pairs showing significant differences in the sprouting test but similar high GI rates in this study could be valuable materials for understanding how those factors influence PHS resistance.

Apart from PHS phenotyping, we also measured other agronomic traits to investigate possible relationships between PHS and these traits. Statistical analysis of the NIL population showed positive correlation between PHS susceptibility and plant height and grain number per spike, two complex quantitative traits in wheat that are strongly influenced by interacting genetic and environmental factors. Numerous studies in the past two decades have identified QTLs related to plant height or yield-related components on all 21 wheat chromosomes [28, 30, 32, 35]. Notably, for both plant height and grain number per spike, consistent QTLs across multiple genetic backgrounds have been identified on chromosome 4BL. The dwarfing gene *Rht-B1,* which can increase grain yield and reduce plant height [29], is located on the short arm of chromosome 4B [43]. The QTL *QPhs.ocs-4B.1* targeted in this study is located on the long arm of chromosome 4B [19], suggesting the possibility of another gene(s) regulating plant height and grain number per spike, as well as PHS resistance.

Some studies have reported negative correlation between plant height and grain number per spike or yield, especially in plants with the dwarfing gene *Rht1* [28, 29, 44]. Other studies have reported highly positive and significant correlations between plant height and grain number per main spike and main spike grain yield [45]. Law et al. [46] identified a positive association between plant height and grain yield within the major dwarfing gene group. In this study, both plant height and grain number per spike decreased in the confirmed resistant isolines, relative to the susceptible isolines, indicating a positive correlation between plant height and grain number per spike and between the two traits and PHS susceptibility. However, there were no apparent differences among the eight pairs of isolines for grain yield. A possible explanation is that the genome region affected not only plant height and grain number, but other yield component traits such as spike number and grain weight, and final yield is determined by a balance of these gene effects [47, 48].

In contrast to traditional backcrossing methods, we used the molecular marker- assisted heterogeneous inbred family (HIF) method to generate NILs [49], assisted by a fast generation cycling system [50, 51]. The HIF method is useful for developing multiple sets of NILs with different genetic backgrounds from a single cross, while the fast generation cycling system dramatically shortens the life-cycle by using young embryo culture and promoting flower differentiation. Combining HIF with the fast generation system and marker-assisted selection enabled the fast development of NILs with multiple genetic backgrounds. This approach has been successful in the development of NILs for some major QTL in wheat [52–54] and barley [55].

This research adopted SNP assays using the wheat 90 K Illumina iSelect chip to detect potential genes responsible for PHS resistance. Eight SNPs with consistent contrasting genotypes across the five confirmed NIL pairs were identified and associated with five candidate genes located on chromosome 4B. Of the five functional genes, *TraesCS4B01G239700* was described as a heat shock transcription factor (Hsf). A study in *Arabidopsis thaliana* indicated that a specialized Hsf, *HsfA9*, exclusively expressed in later stages of seed development, coincided with the acquisition of dormancy, and response to abscisic acid [38]. Almoguera et al. [39] detected *HsfA9* expressed in embryos, but it disappeared shortly after seed germination in sunflower. In addition, *HsfA9* had minor effects on seed maturation and germination [53]. Another candidate gene *TraesCS4B02G247900* was described as an ATP-dependent RNA helicase. Recently, a study identified a DEAD-box helicase ATP-binding protein from rice in the response to ABA stress, which is the main plant hormone that regulates germination [41]. A study on DEAD-box RNA helicase showed its influence on germination at high temperature in *Arabidopsis* seeds [42]*.* The other three genes were reportedly related to other functions. Gene *TraesCS4B02G253300* is an α-L-fucosidase two related gene, involved in xyloglucan metabolism in *Arabidopsis* [56]. Gene *TraesCS4B02G217800* reportedly encodes a myosin-binding protein drive endomembrane trafficking and cytoplasmic streaming [57], gene *TraesCS4B02G229600* is involved with plant defense responses to fungi or bacteria in *Arabidopsis* [58]. In wheat, although there are no reports on the function of *TraesCS4B01G239700* and *TraesCS4B02G247900* on germination, the results from this study suggest that further research could elucidate the influence of these genes on wheat PHS and seed dormancy performances. Also, the identified SNPs will be further evaluated in larger populations of commercial wheat genotypes to confirm their usefulness in wheat breeding programs.

## Conclusions

The NILs developed and validated in this study confirmed the importance of *QPhs.ocs-4B.1* for PHS resistance. The confirmed NILs together with the candidate genes identified by the SNP assay are valuable materials/information for future studies in fine mapping, molecular marker development and the final cloning of 4BL genes responsible for PHS resistance.

## Methods

### Plant materials

NIL pairs were developed from two biparental populations including 200 F_2_ plants of Chara × DM5637B*8 and 40 F_2_ plants of SUN326AE × Westonia. DM5637B*8 and SUN326AE are the two PHS resistance genotypes, whereas Westonia and Chara are the two Australian non-dormant, PHS susceptible varieties [18, 22, 59, 60]. Seeds of the four parents were obtained from Australian Grains Genebank, Horsham, Victoria, Australia. Molecular marker-assisted HIF method [49], in combination with a fast generation cycling system [50] was used to develop NILs from these two segregating populations. Heterozygous progenies identified from marker-assisted selection (as described in the below “Molecular marker analysis” section) were selfed for the next generation. In detail, 240 F_2_ lines were planted in the glasshouse. Around 10 days after anthesis, young embryos from each plant were germinated on an artificial medium for next generation [50]. At least four young seedlings from each plant were transferred into pot for further growing. Only heterozygous progenies, identified using marker-assisted selection at three-leaf stage, were kept to grow into next generation for the next round of selection. This heterozygous progeny selection and selfing progress was continued until the F_7_ generation. For F_8_ individuals, either homozygous with the resistant allele from SUN326AE or DM5637B*8, or with the susceptible allele from Westonia or Chara, were identified from the progenies of F_7_ heterozygous plants as putative NIL pairs. Seeds from these putative NIL pairs were multiplied, and their PHS responses were assessed to identify the true NILs. The two populations were grown in controlled environments in tissue culture rooms and glasshouses at The University of Western Australian in Perth, with DNA marker-assisted selection in each generation.

### Molecular marker analysis

Genomic DNA was isolated from two-week-old seedlings using a modified CTAB method [61] for each plant in every generation. SSR marker *Xgwm495* (Forward: GAGAGCCTCGCGAAATATAGG; Reverse: TGCTTCTGGTGTTCCTTCG), located close to *QPhs.ocs-4B.1*, was used to identify heterozygous progeny from the two populations. PCR reactions were performed and amplification products were viewed following the protocol described in Wang et al. [35].

### Sprouting test

At physiological maturity, as the head and the peduncle lost their green color (about 30 days after flowering), four spikes from each plant were harvested for PHS assessment. Three plants as three replicates for each putative isoline were tested. The spikes were wrapped in moist filter paper immediately after being immersed in distilled water for 6 h, and then randomly arranged standing upright in a sealed box with 100% humidity for 7 days. The box was placed in a controlled temperature room with a constant condition of 22 °C and 16 h light/8 h dark. After 7 days, the spikes were immediately dried at 65 °C to prevent further germination and then gently threshed to collect grains, which were examined for symptoms of sprouting damage (breakage of the seed coat near the embryo). The sprouting percentage was calculated as the percentage of sprouted grains to the total number of grains, as described in Shorinola et al. [62].

### Germination index assay

GI assay was conducted on all putative pairs of the isolines. Seeds for the GI assay were harvested at the same time as the spikes for the sprouting test, and gently threshed to keep uniform seed condition. Three replicates were used for each genotype. For each replicate, 50 healthy seeds were evenly distributed in a 90 mm Petri dish with deionized water and filter paper for 7 days. The Petri dish were sealed with Parafilm to prevent water evaporation. Germinated seeds were counted daily and then removed. The germination index was calculated as follows [63]:

$$ \mathrm{GI}=\frac{7\times {n}_1+6\times {n}_2+5\times {n}_3+4\times {n}_3+\dots +1\times {n}_7}{7\times \mathrm{total}\kern0.17em \mathrm{number}\kern0.17em \mathrm{of}\kern0.17em \mathrm{grains}} $$

where n1, n2,…, n7 are the number of seeds germinated on the first, second, and subsequent days until the 7th day, respectively. The maximum index is 1.0 if all grains germinate on the first day.

### Investigation of the correlation between PHS resistance and other agronomic traits

To investigate possible correlations of other agronomic traits with PHS resistance, measurements of various traits were taken during plant growth, including days to flowering, days to physiological maturity, plant height, plant dry matter, spike number, grain per spike, yield per plant, and 1000-kernel weight. Days to flowering for each plant was recorded as the date when half of the spikes were flowered; days to physiological maturity was recorded as the head and the peduncle lost their green color. Plant height was measured from the ground to the tip of the tallest tiller of the plant. Plant dry matter was determined after the plants were harvested and oven dried at 80 °C to constant weight; and then spike number, grain per spike, yield per plant and 1000-kernel weight were measured for each plant.

### Statistical analysis

Statistical analysis was performed using GenStat statistical software 17th edition. The sprouting percentages from each putative NIL pairs were analysed using the Student’s t-test to determine any significant differences between the isoline pairs with or without the resistant allele from the resistant genotypes SUN326AE and DM5637B*8. Correlation analysis was conducted to investigate possible morphological traits correlated with PHS resistance.

### 90 K SNP genotyping and candidate gene identification

Genomic DNA samples of the confirmed NILs were genotyped by the custom Illumina wheat 90 K iSelect Assay [37]. SNP clustering and genotype calling were performed using GenomeStudio 2.0 software (Illumina). SNPs with a call frequency < 0.8 (i.e. missing data points > 20%) or minor allele frequency (MAF) < 0.05 and SNPs with > 0.25 heterozygous calls were removed. The sequences of the SNPs that differed between the NIL pairs were used to perform a BLAST search against the wheat reference genome [64]. The SNPs located on the 4BL chromosome arm, especially those near markers *Xgwm495* and *Xgwm375*, were scrutinized for candidate genes underlying the targeted QTL.

## Data Availability

The datasets used and/or analysed during the current study are available from the corresponding author on reasonable request.

## Abbreviations

GI: Germination index
HIF: Heterogeneous inbred family
Hsf: Heat shock transcription factor
NIL: Near-isogenic line
PHS: Pre-harvest sprouting
QTL: Quantitative trait locus/loci
SNP: Single nucleotide polymorphism
